# Self-assembly of octadecyltrichlorosilane: Surface structures formed using different protocols of particle lithography

**DOI:** 10.3762/bjnano.3.12

**Published:** 2012-02-09

**Authors:** ChaMarra K Saner, Kathie L Lusker, Zorabel M LeJeune, Wilson K Serem, Jayne C Garno

**Affiliations:** 1 Chemistry Department, Louisiana State University, 232 Choppin Hall, Baton Rouge, LA 70803, USA. Telephone: +1 225 578 8942; Fax: +1 225 578 3458

**Keywords:** atomic force microscopy, nanopatterning, nanostructures, octadecyltrichlorosilane, particle lithography, self-assembled monolayer, self-assembly

## Abstract

Particle lithography offers generic capabilities for the high-throughput fabrication of nanopatterns from organosilane self-assembled monolayers, which offers the opportunity to study surface-based chemical reactions at the molecular level. Nanopatterns of octadecyltrichlorosilane (OTS) were prepared on surfaces of Si(111) using designed protocols of particle lithography combined with either vapor deposition, immersion, or contact printing. Changing the physical approaches for applying molecules to masked surfaces produced OTS nanostructures with different shapes and heights. Ring nanostructures, nanodots and uncovered pores of OTS were prepared using three protocols, with OTS surface coverage ranging from 10% to 85%. Thickness measurements from AFM cursor profiles were used to evaluate the orientation and density of the OTS nanostructures. Differences in the thickness and morphology of the OTS nanostructures are disclosed based on atomic force microscopy (AFM) images. Images of OTS nanostructures prepared on Si(111) that were generated by the different approaches provide insight into the self-assembly mechanism of OTS, and particularly into the role of water and solvents in hydrolysis and silanation.

## Introduction

Self-assembled monolayers (SAMs) of organosilanes have become important as surface resists and functional coatings for micro- and nanopatterning applications [1–9]. The surface self-assembly of organosilanes such as octadecyltrichlorosilane (OTS) is complicated, with multiple steps of hydrolysis, cross-linking and silanation [10–13]. To develop robust and reproducible lithography procedures with OTS, parameters, such as temperature, humidity, solvents, physical deposition conditions, and mask materials, can be systematically changed to enable nanoscale studies of surface assembly.

For methods of particle lithography, a surface mask of polystyrene latex or silica mesospheres is used to direct the deposition of organic thin films and nanomaterials. The surface density of nanostructures can be designed by selecting the diameter of mesospheres, for high-throughput patterning on the order of 10^9^ nanostructures per square centimeter. Different approaches with particle lithography have been successful for producing periodic, 2D arrays of nanostructures of different materials and molecular films, including metals [14–15], nanoparticles [16–19], proteins [20–22], polymers [23–26] and SAMs [27–31]. A significant advantage of using organosilanes in comparison to thiolated SAMs is that silane films can be prepared on a wide range of substrates, such as glass [32], mica [33–35], quartz [36–37], indium tin oxide (ITO) [38], or silicon (Si) [11,32,39–42] or metal oxides such as gold [43–44]. This versatility of organosilanes in the preparation of nanostructures on different surfaces will be helpful for new applications and developments in the patterning of biomolecules or nanoparticles for optical measurements and biosensor surfaces.

The morphology of SAMs or nanostructures of OTS reflects a balance of the interactions that occur between the silane precursor and the silanol groups, interactions between the end groups, interactions between the alkyl chains of the silane molecules, and the nature of the substrates [45–46]. These intramolecular interactions, along with parameters such as temperature, solvent type and trace amounts of water, present a challenge for reproducible fabrication with organosilanes such as OTS [10–11,45–50]. Preparation methods affect the growth rate, surface coverage and orientation of OTS [51].

Molecular-level differences in the thickness and morphology of OTS nanostructures prepared by different lithography procedures can be investigated by performing atomic force microscopy (AFM) studies [52–53]. Particle lithography enables control of the deposition parameters for tailoring the surface coverage, surface geometries and pattern dimensions. Close-packed arrays of latex or silica mesoparticles were used as surface masks to direct the deposition of OTS on surfaces to form nanopatterns. Essentially, the physical state of the molecule was changed for the three protocols. Molecules were applied either in a vapor phase, as a liquid film, or under dilute-solvent conditions, to enable nanoscale studies of the surface organization and self-assembly of OTS.

## Results and Discussion

**Figure 1:**
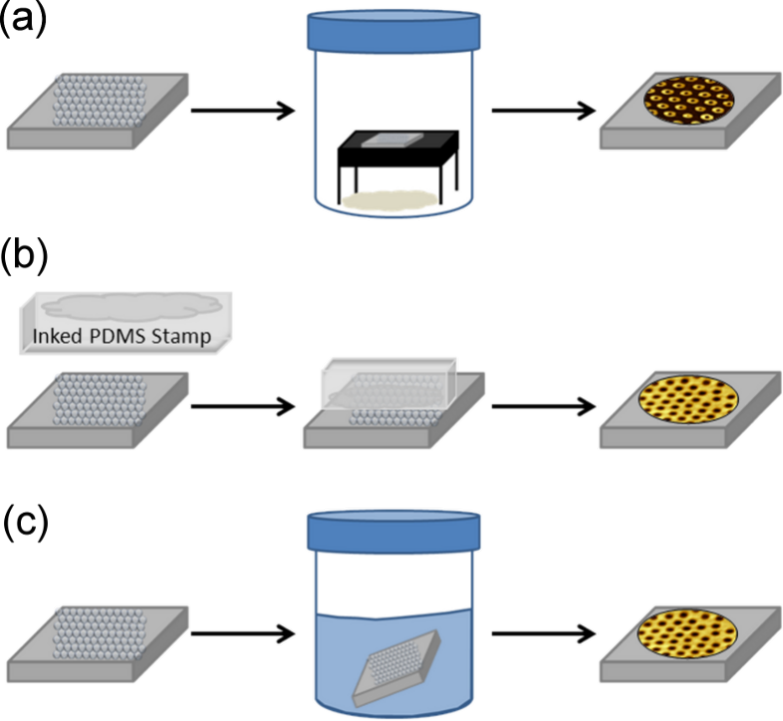
Strategies for preparing organosilane nanostructures by means of particle lithography. Basic steps are shown for (a) vapor deposition; (b) contact printing with PDMS; and (c) solution immersion of Si(111) surfaces coated with mesoparticle masks.

A comparison of the geometries and thicknesses of the nanostructures produced by particle lithography was used to systematically investigate parameters for surface self-assembly of octadecyltrichlorosilane (OTS). Three methods of particle lithography for preparing organosilane nanostructures are compared, as shown in Figure 1. Each approach uses a different strategy for applying the organosilanes to the masked surface of Si(111), using either heated-vapor deposition, contact printing, or immersion in a silane solution. For comparison of the different particle lithography strategies, the samples were prepared using masks of polystyrene latex (200 nm diameter); the mesospheres have a size variation of 1–2%. Organosilanes attach to the surfaces by successive steps of hydrolysis and condensation, therefore nanoscopic amounts of water are needed to initiate the reaction. By controlling the drying parameters of the latex masks, different nanopattern geometries are produced [30,38].

### Nanostructures produced by particle lithography using vapor deposition of OTS

**Figure 2:**
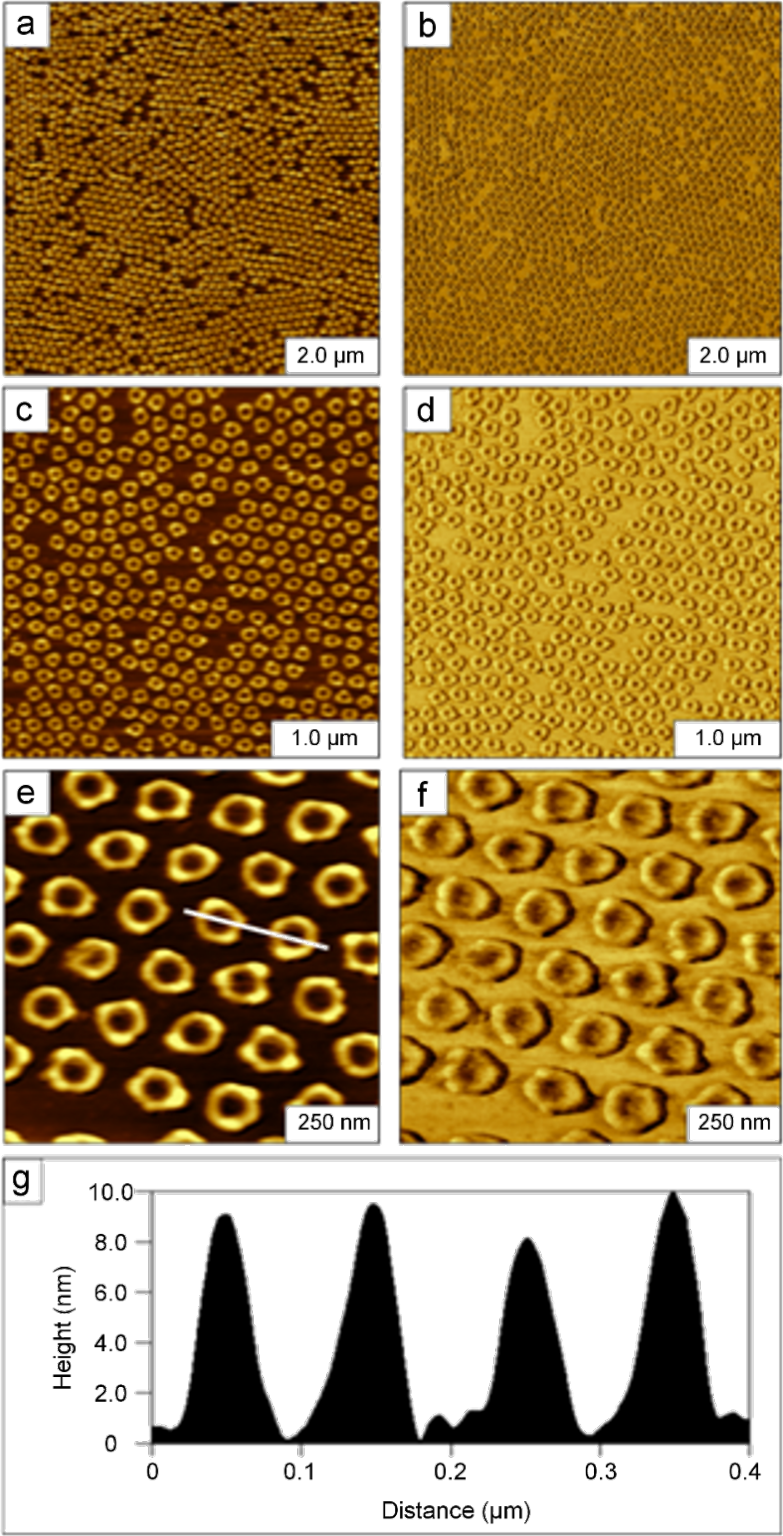
Combining particle lithography with vapor deposition of OTS produced ring-shaped nanostructures. (a) Contact-mode topograph, 8 × 8 µm^2^; (b) simultaneously acquired lateral-force image. (c) Higher-magnification topograph (4 × 4 µm^2^); (d) corresponding lateral-force image. (e) zoom-in topography view of 1 × 1 µm^2^ area; and (f) lateral-force frame. (g) Height profile for the white line cross-section in (e).

By combining particle lithography with vapor deposition of OTS, arrays of ring-shaped nanostructures were formed on Si(111), as shown in the contact-mode AFM images in Figure 2. A wide-area frame (8 × 8 µm^2^) in Figure 2a and Figure 2b reveals the arrangement of hundreds of circular nanostructures, showing a few gaps corresponding to the uncovered substrate. There are 336 ring nanostructures within the 4 × 4 µm^2^ frame of Figure 2c and Figure 2d. If the array were perfectly ordered and densely packed the frame would accommodate 360 nanostructures, indicating a defect density of ~7%. The dimensions and circular shapes of the nanostructures correspond to highly regular circles of consistent heights. Within the 1 × 1 µm^2^ close-up view, 29 patterns are packed closely together (Figure 2e and Figure 2f). This scales to an overall surface density of 3 × 10^9^ patterns/cm^2^. The areas confined within the centers of the rings appear to have the same contrast as the surrounding substrate for both the topography and lateral-force frames of Figure 2e and Figure 2f. Careful examination of zoom views from this experiment shows discontinuous surface coverage of small OTS islands with molecular heights of ~0.5 nm. The central areas of the rings were masked by the latex mesospheres, and meniscus-shaped areas of OTS were formed surrounding the base of the latex particles, generating the nanopatterns. The cursor line profile across two of the rings (Figure 2g) shows that the baseline within the rings is nearly the same height as the background areas of bare Si(111). The thickness of OTS monolayers has been reported to range from 2.26 to 2.76 nm under various conditions of sample preparation [1,42,54–56]. An “ideal” OTS monolayer of a dense, highly ordered film, in which all of the molecular tails are fully extended and oriented perpendicular to the substrate, would have a well-defined thickness of 2.6 ± 0.1 nm. The height of the rings is measured as 10 ± 2 nm, which corresponds to 3–4 layers of OTS (Figure 2). The center-to-center spacing between the ring structures is approximately 200 nm, which matches the diameter of the latex mask.

**Figure 3:**
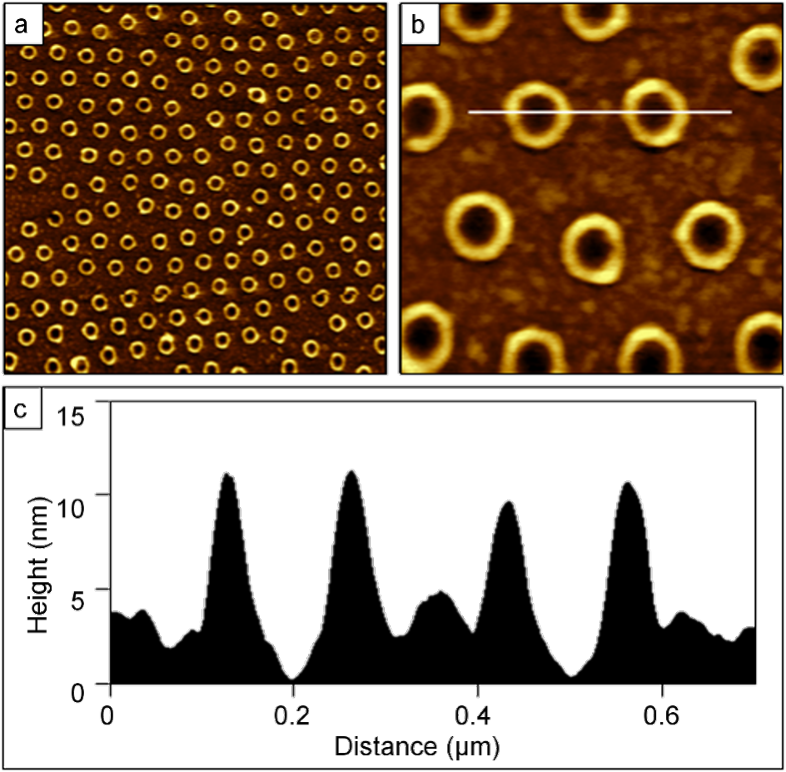
Particle lithography with vapor deposition of OTS produced multilayered ring nanostructures surrounded by an OTS monolayer. (a) Contact-mode topograph, 4 × 4 µm^2^; (b) zoom-in view, 1 × 1 µm^2^; (c) corresponding cursor profile for (b).

When the latex masks were dried, a water meniscus persisted at the base of each latex sphere on the surface, and this defined the reaction sites for hydrolysis and condensation of the organosilanes [54]. For the example in Figure 2, the interstitial areas between the OTS rings do not have consistent coverage, and OTS was shown to bind mainly in the areas pinned beneath the base of latex spheres. The cursor profile shows that the areas surrounding the rings and inside the rings are nearly the same height, where the height scale refers to the baseline of the uncoated substrate. The location of water residues on the surface defines the sites for OTS binding; for example, with the more hydrophilic substrate of mica (0001) attachment to the interstitial areas of the surface between spheres was observed for latex masks that were briefly dried [57]. If the masks formed on Si(111) are dried briefly, more water persists on the surface, thus OTS also binds to the interstitial areas between the rings (Figure 3). An example is shown of OTS nanopatterns with different heights outside and within the rings. The cursor profile across two of the ring patterns shows a height of 4 ± 1 nm between the rings, the rings measure 12 ± 2 nm in height, and the shallowest area inside the rings can be used as a reference baseline for the uncoated Si(111) substrate. Water residues persist across the surface; however, there is a higher zone of water trapped in the meniscus areas surrounding the spheres. Interestingly, we have observed that the height of the meniscus is greater for larger-diameter latex spheres, which correspondingly leads to scalable heights for organosilane-ring nanopatterns [54].

### Particle lithography combined with contact printing with PDMS stamps

**Figure 4:**
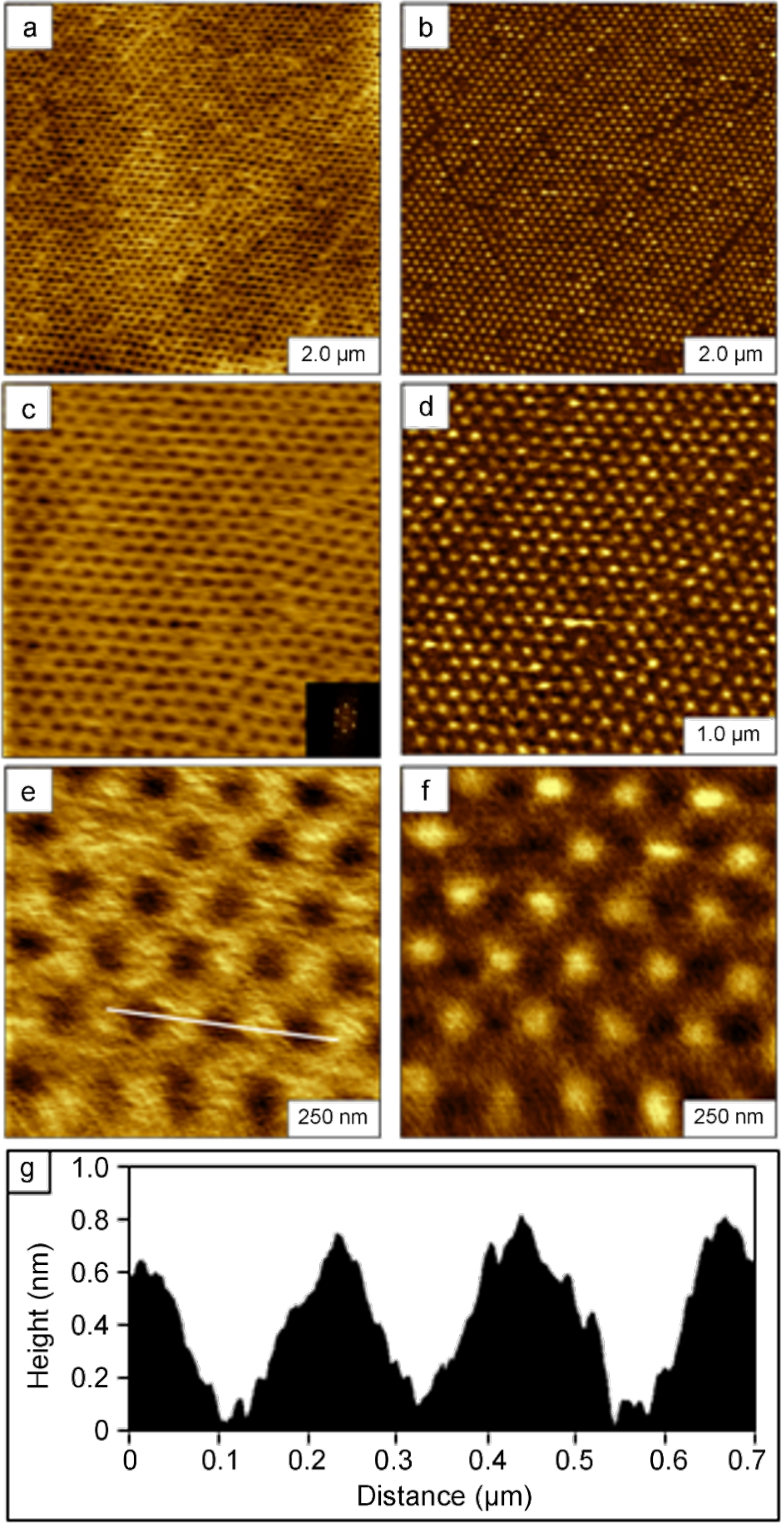
Nanopore structures of OTS were formed with particle lithography combined with contact printing. Contact-mode AFM images are shown for a sample prepared with 200 nm latex mesospheres on Si(111). (a) 8 × 8 µm^2^ topograph and (b) corresponding lateral-force image. (c) Zoom-in topograph (4 × 4 µm^2^) with FFT shown in the inset; (d) simultaneously acquired lateral-force frame. (e) Topography frame (1 × 1 µm^2^) with (f) showing the corresponding lateral-force image. (g) Height profile for the white line in (e).

To produce monolayer nanostructures of OTS, particle lithography with contact printing and immersion were evaluated to optimize the deposition conditions for achieving a densely packed SAM. Images of a nanostructured film of OTS prepared by using particle lithography combined with contact printing are shown in Figure 4. A honeycomb arrangement of nanopores is shown in Figure 4a, with approximately 25 × 20 rows of dark holes within a film of OTS within the frame. The corresponding lateral-force image of Figure 4b reveals the shapes of the holes as bright spots, corresponding to the bare areas of Si(111) where latex was displaced. At higher magnification, 438 nanopores are packed within the 4 × 4 µm^2^ images of Figure 4c and Figure 4d, which scales to an approximate surface density of 2.7 × 10^9^ nanostructures/cm^2^. This value is comparable to the pattern density for Figure 2, because the latex diameter of the surface mask determines the packing density. The inset of Figure 4c is an FFT of the topograph, and represents a mathematical average of the 2D lattice of the hexagonal array. A further magnified view is presented in Figure 4e and Figure 4f showing ~27 nanopores. The lateral-force image confirms that the holes are uncovered Si(111), evidenced by the distinct change in chemical contrast between OTS and the nanopores. Referencing the uncovered areas of the substrate as a baseline, the height of the OTS film measures 0.6 ± 0.1 nm (Figure 4g), which indicates submonolayer surface coverage. Since the overall diameter of an alkyl chain is approximately 0.5 nm, the thickness value suggests a side-on arrangement of the molecules, with the backbone of the molecule oriented parallel to the substrate.

Multiple replicate samples were prepared using contact printing, for different size masks, showing that the heights were consistent with the example of Figure 4. For OTS transfer by contact printing, a solution of solvent and silane at a 40% (v/v) concentration was placed on the surface of a PDMS block and dried. This process most likely forms a thin cross-linked film of OTS that does not bind to the polymeric surface of PDMS. After the mask was placed in contact with the sample, the liquid film was transferred to the Si(111) substrate by liquid permeation through the latex mask.

### Particle lithography by immersion of latex-masked substrates in silane solutions

**Figure 5:**
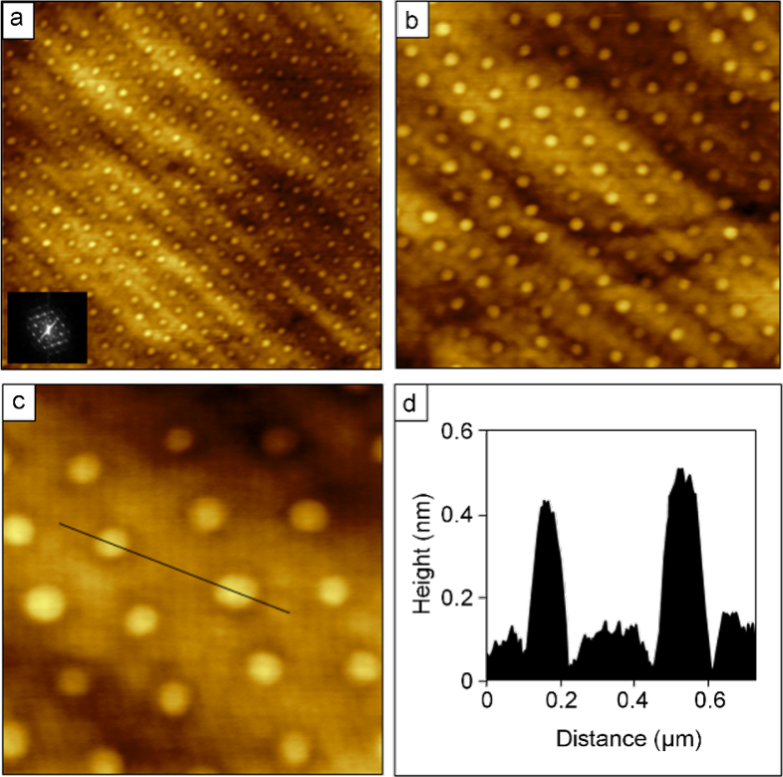
Nanodots of OTS produced with immersion of annealed latex masks. Contact-mode AFM images are shown for OTS nanostructures formed on Si(111) with 200 nm latex. (a) Topography image, 4.5 × 4.5 µm^2^ and FFT inset; (b) zoom-in, 2.5 × 2.5 µm^2^; (c) close-up view, 1 × 1 µm^2^; (d) height profile of the line in (c).

A completely different morphology other than rings or nanopores was observed for OTS nanostructures produced by the immersion of particle masks. Dot-shaped nanostructures were produced by using latex-particle lithography with immersion, as shown in Figure 5 with wide-area and zoom-in topography views. The long-range periodicity of the array of nanodots is shown with an FFT within the inset of Figure 5a. The surface density of the nanodots is approximately 3.3 × 10^9^ nanostructures/cm^2^, showing ~120 nanopatterns within the 2.5 × 2.5 µm^2^ frame shown in Figure 5b. The heights of the nanodots measure 0.5 ± 0.3 nm.

Immersion of a masked substrate in a solvent is the most common approach for preparing films of OTS, and has produced the most consistent thickness of a monolayer. However, immersion in solvents causes rapid detachment of the latex masks. To enable an immersion process for particle lithography, a brief heating step was developed to solder the latex beads to the substrate (75 °C for 30 min). Latex deforms when heated, leaving less surface area available for OTS deposition [58]. After the heating step, the only remaining areas that were not masked by latex were the triple-hollow sites formed between spheres, and the geometries and periodicity of the nanodots shown in Figure 5 correspond to these sites.

### Surface masks of colloidal silica mesospheres

**Figure 6:**
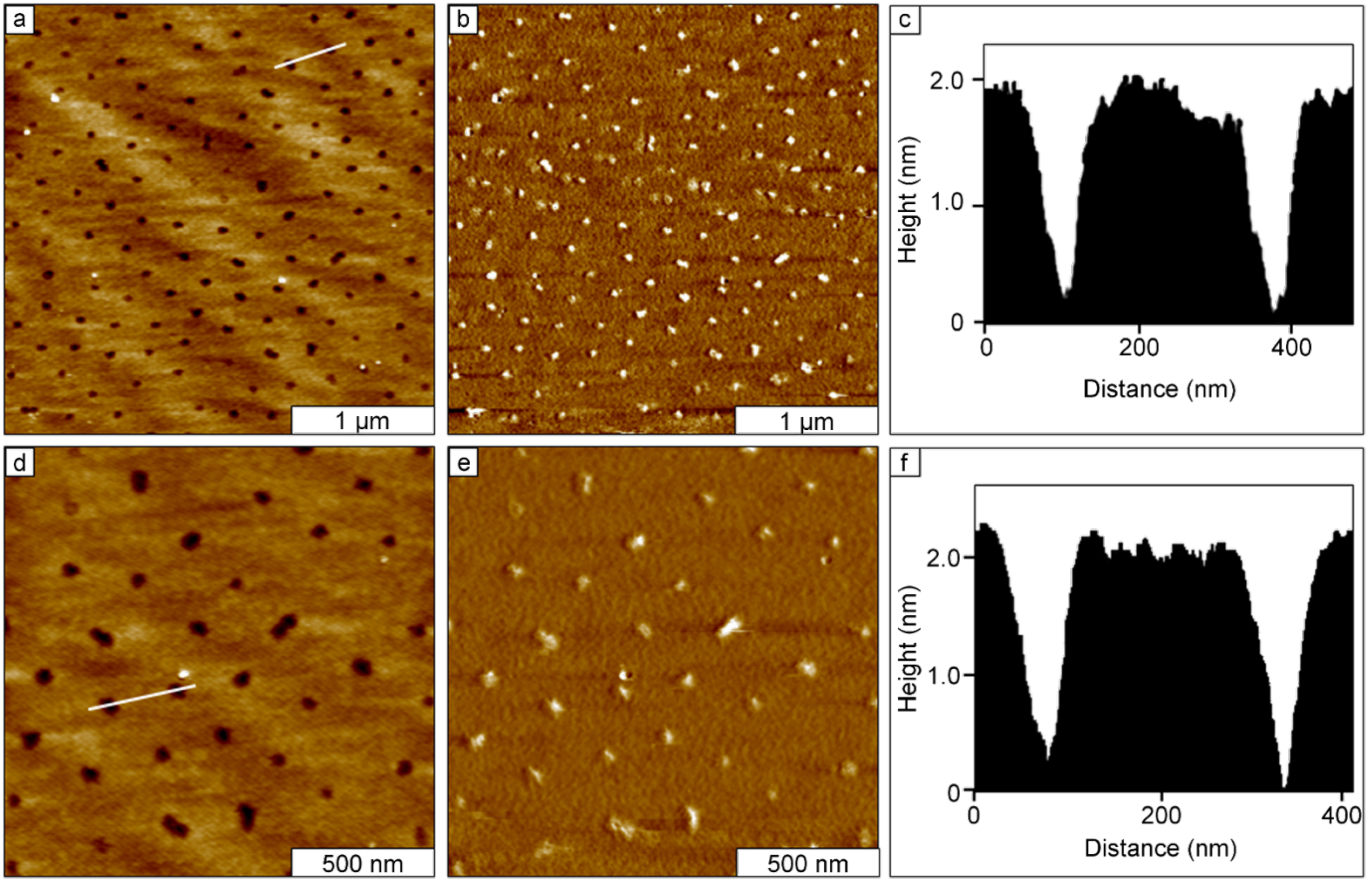
Nanostructured film of OTS produced by immersion of annealed silica masks in OTS solutions. Contact-mode AFM images are shown for OTS nanostructures formed on Si(111) with 250 nm silica mesospheres: (a) 2.75 × 2.75 µm^2^ topograph; (b) corresponding lateral-force view; (c) height profile of the line in (a); (d) 1.5 × 1.5 µm^2^ zoom-in view of (a); (e) lateral-force frame simultaneously acquired with (d); (f) cursor plot for the line in (d).

Silica mesospheres do not deform as readily as polystyrene latex, and can sustain longer heating at higher temperatures [28]. The results for OTS nanostructures produced with silica masks are shown in Figure 6. Nanohole structures are shown in the wide-area (Figure 6a; 2.75 × 2.75 µm^2^) and high-magnification images (Figure 6d; 1.5 × 1.5 µm^2^).The topography frames reveal periodic patterns within a monolayer film of OTS, with exquisitely small holes at the locations where silica mesospheres (250 nm diameter) were displaced. There are 38 nanopores in the zoom-in views of Figure 6d and Figure 6e, which would scale to a surface density of 1.7 × 10^9^ patterns/cm^2^. The depth of the OTS film was measured to be 2.0 ± 0.2 nm (Figure 6c and Figure 6f) referring to the uncovered area of Si(111) as the baseline. This value corresponds to a nearly upright configuration of an OTS monolayer. The diameters of the nanoholes were measured to be 102 ± 11 nm. The center-to-center spacing between the holes corresponds to the diameters of the silica mesospheres (250 nm) used as a structural template to pattern the OTS. The overall coverage of the OTS film was estimated to be ~85% of the surface.

### Molecular orientation of OTS within nanopatterns

For the three approaches described, the procedures are highly reproducible. Multiple samples were prepared and formed consistent shapes and thicknesses, as summarized in Table 1. A cross-linked multilayer was formed for rings of OTS, with different thicknesses within the interstitial areas of the substrates between the rings (Figure 2 and Figure 3). Using the contact-printing approach with PDMS stamps, the thickness of the OTS film corresponds to submonolayer surface coverage (Figure 4). Despite multiple tests and samples, a monolayer thickness was not achieved with latex masks and contact printing of OTS. A similar height was produced by using the immersion of annealed latex masks. The brief annealing step was effective for producing exquisitely small areas on the surface for the preparation of nanodot structures; however, the heights do not correspond to an upright orientation of OTS (Figure 5). For evaluating the molecular orientation, the thickness measurements of OTS films were obtained exclusively from AFM height profiles, rather than spatially averaged results from infrared spectroscopy. The theoretical thickness for a side-on orientation of OTS with the backbone oriented parallel to the substrate would measure 0.5 ± 0.1 nm. By changing to silica mesospheres for the immersion strategy, a taller OTS film was produced than that observed for the latex masks (Figure 6). This new result suggests that the nature of the surface of the mesosphere masks can affect the outcome of patterning with particle lithography. Polystyrene latex has been described as a “hairy” particle, with strands of polystyrene extending across the exterior surface areas of the beads. The strands provide surface sites for interaction with OTS to produce a cross-linked arrangement within the nanodot surface structures. The consistent and reproducible geometries of the different OTS nanostructures are not necessarily a “failed” approach for particle lithography, rather a range of different surface shapes and thicknesses can be generated for selected applications. Overall, the highest-quality monolayer of OTS was produced by using the immersion of annealed mesosphere masks of silica.

**Table 1:** Particle lithography with OTS based on different approaches for surface deposition.

method	mask	nanostructure shape	surface coverage (OTS)	OTS thickness

vapor deposition	200 nm latex	ring nanostructures of OTS multilayers	40%	10 ± 2 nm
contact printing	200 nm latex	nanopores of uncovered substrate within an OTS film	26%	0.6 ± 0.1 nm
immersion of annealed latex masks	200 nm latex	nanodots	10%	0.5 ± 0.3 nm
immersion of annealed silica masks	250 nm silica	nanopores of uncovered substrate within an OTS monolayer	85%	2.0 ± 0.2 nm

## Conclusion

The surface self-assembly of OTS was studied by using approaches of particle lithography combined with vapor deposition, contact printing and immersion. By changing the physical approaches for applying molecules to surfaces, the molecular arrangement and surface density can be controlled. For example, submonolayer surface coverage was obtained by using protocols with contact printing. Changing the material composition of the mesoparticle masks produced entirely different surface structures for annealed masks of latex and silica spheres. The meniscus sites of water residues at the base of latex spheres furnish local containers for self-polymerization reactions to generate multilayer surface structures. Optimized structures with nearly the thickness of an ideal monolayer were achieved by using annealed masks of colloidal silica mesospheres immersed in OTS solutions. Further experiments are in progress to directly compare the surface structures formed based on immersion protocols with latex and silica masks.

## Experimental

**Atomic force microscopy (AFM)**. Organosilane thin films were characterized using models 5420 and 5500 scanning probe microscopes operated in contact or tapping-mode AFM. (Agilent Technologies, Chandler, AZ). Lateral force images were acquired for either the trace or retrace views corresponding to the scan direction of the selected topography frames. The color scales of lateral-force images indicate differences in tip–surface interactions, but were not normalized for the comparison of friction changes between different tips or experiments. The tips were silicon nitride probes. Tips used with tapping-mode AFM were rectangular shaped ultrasharp silicon tips that have an aluminium reflex coating, with a spring constant of 48 N/m (Nanoscience Instruments, Phoenix, AZ). For contact-mode images, V-shaped tips (Veeco Probes, Santa Barbara, CA) with an average force constant of 0.5 N/m were used. Data files were processed by using Gwyddion open-source software, which is freely available on the internet and supported by the Czech Metrology Institute [59]. Estimates of surface coverage were obtained for individual topography frames by manually converting images to black and white using thresholding and pixel counting with UTHSCA Image Tool [60].

**Preparation of latex-particle masks**. Polished silicon wafers doped with boron (Virginia Semiconductor, Fredericksburg, VA) were used as substrates. Pieces of Si(111) were cleaned by immersion in a 3:1 (v/v) piranha solution for 1 h. Piranha solution consists of sulfuric acid and hydrogen peroxide, which is highly corrosive, and should be handled carefully. After acid cleaning, the substrates were rinsed with copious amounts of deionized water and dried in air. Size-sorted, monodisperse polystyrene latex mesospheres (200 nm diameter) were used as surface masks for patterning (Thermo-Fisher Scientific, Waltman, MA). Aqueous solutions of latex were cleaned by centrifugation to remove surfactants or contaminants. Approximately 300 µL of the latex solution was placed into a microcentrifuge tube and centrifuged at 15,000 rpm for 15 min. A solid pellet was formed, and the supernatant was removed and replaced with deionized water. The latex pellet was resuspended with 300 µL of deionized water by vortex mixing to prepare a 1% w/v solution. The washing process was repeated twice. A drop (10–15 µL) of the cleaned mesospheres was deposited onto clean Si(111) substrates and dried under ambient conditions (25 °C, ~50% relative humidity) for at least one hour, in order to form surface masks for nanolithography.

**Particle lithography combined with vapor deposition**. The masked substrates were placed into sealed glass vessels for vapor deposition of organosilane. The samples were placed on a raised platform in a jar containing 300 µL of neat octadecyltrichlorosilane (Gelest, Morrisville, PA). A vapor was generated by heating the vessel in an oven at 70 °C. After at least 6 h, the samples were removed and rinsed with ethanol and water to remove the latex masks.

**Particle lithography with contact printing**. For contact printing, an inked block of polydimethylsiloxane (PDMS) (Sylgard 184, Dow Corning) was used to transfer OTS to the substrate through a physical mask of latex spheres. A drop (10–12 µL) of an OTS solution in bicyclohexyl was deposited onto a clean, dry block of PDMS (2 × 2 cm^2^). A 30 µL volume of a 40% v/v solution of OTS in bicyclohexyl was deposited and spread evenly over the PDMS block, then quickly dried in a stream of ultra-high-purity argon. The PDMS block coated with OTS was placed on top of the masked substrate. The film of OTS was transferred from the PDMS block through the latex mask to the substrate by permeation. The areas of the Si(111) surface located directly underneath the latex particles were protected from silane deposition. After 1 h of physical contact, the PDMS block was removed. The sample was rinsed with copious amounts of deionized water. In the final step, the mask of latex particles was cleanly removed by sonication and rinsing with ethanol and deionized water. After removal of the mask, a nanostructured film of OTS was generated on the surface.

**Particle lithography with immersion**. For the immersion strategy of particle lithography, the masked substrates of latex were heated for 30 min at 75 °C in order to anneal the beads to the surface. Masked substrates of colloidal silica mesospheres were heated for 12 h at 140 °C. After heating, the samples were cooled for at least 20 min under ambient conditions. The mesosphere-coated substrates were then immersed into a 0.1% solution of OTS in bicyclohexyl or anhydrous toluene for 1 h. Next, the samples were removed and rinsed with ethanol and deionized water, and sonication was used to remove the latex masks.
